# Real-time energy consumption and air pollution emission during the transpacific crossing of a container ship

**DOI:** 10.1038/s41598-022-19605-7

**Published:** 2022-09-10

**Authors:** Chin-Ko Yeh, Chitsan Lin, Hsueh-Chen Shen, Nicholas Kiprotich Cheruiyot, Duy-Hieu Nguyen, Chi-Chung Chang

**Affiliations:** 1 Ph.D. Program in Maritime Science and Technology, National Kaohsiung University of Science and Technology, Kaohsiung, 81157 Taiwan, ROC; 2 Department of Marine Environmental Engineering, National Kaohsiung University of Science and Technology, Kaohsiung, 81157 Taiwan, ROC; 3 Super Micro Mass Research and Technology Center, Cheng Shiu University, Kaohsiung, 833301 Taiwan, ROC; 4 Center for Environmental Toxin and Emerging-Contaminant Research, Cheng Shiu University, Kaohsiung, 833301 Taiwan, ROC; 5 Department of Construction Management, College of Technology, University of Houston, Houston, Texas 77004 United States

**Keywords:** Environmental sciences, Ocean sciences

## Abstract

This study presents the real-time energy consumption of a container ship’s generator engine on two round-trips from the West Coast of the US to the East Asian ports and analyzes the ship’s PM_10_, PM_2.5_, NO_x_, SO_x_, CO, and HC emissions, shore power usage, and factors affecting energy consumption. The average total energy consumption and air emissions for the two round trips were 1.72 GWh and 42.1 tons, respectively. The transpacific crossing segment had the highest average energy consumption (2848 ± 361 kWh) and pollutant emission rate (78.9 ± 10.0 kg h^−1^). On the other hand, the West Coast of the US had the least energy consumption due to shore power adoption. Furthermore, switching from heavy fuel oil (HFO) to ultra-low-sulfur fuel oil (ULSFO) greatly reduced the emissions of PM and SO_x_ by > 96% and NO_x_ by 17.0%. However, CO and HC increased by 16.9% and 36.1%, respectively, implying incomplete combustion. In addition, the energy consumption was influenced by the number of reefers and wind. Therefore, this study recommends further research on energy-efficient reefers, generator engine optimization, and shore power adoption to reduce emissions from container ships.

## Introduction

Freight transport by ship is one of the primary drivers of economic globalization and accounts for over 80% of global trade^1^. However, the industry is a major source of water and air pollution and has been directly linked to marine and coastal environmental deterioration^2–7^. Furthermore, international shipping was the third-highest anthropogenic sulfur oxides (SO_x_) emitter globally, after power plants and industry^8,9^. The International Maritime Organization (IMO) reported that ships emit 10.6 and 18.6 million tons of SO_x_ and nitrogen oxides (NO_x_) in a year, respectively^10^. In addition, Gössling, et al.^11^ projected the emissions to triple between 2020 and 2050.

The IMO and regional environmental bodies, e.g., the California Air Resource Board (CARB), have responded by putting in place laws and regulations to prevent the pollution caused by the shipping industry^12,13^. According to the IMO Marine Pollution Convention (MARPOL) Annex VI regulations, ships sailing in Sulfur Emission Control Areas (SECA) must use 0.1% m/m ultra-low sulfur oil (ULSFO)^14,15^. Since January 1, 2020, ships outside SECA are required to use < 0.5% m/m low sulfur fuel^16^. In Californian ports, ships must also abide by the stricter CARB Ocean-Going Ship Fuel Regulations requiring ships to use 0.1% ULSFO 24 nautical miles from California’s coastline. These regulations also do not permit using high-sulfur fuel and scrubbers as an alternative approach^17^.

Low-sulfur fuels have significantly reduced sulfur oxides (SO_x_) and particulate matter (PM) emissions from ships. NO_x_ emissions have also slightly reduced with the switching to lower-sulfur fuels. For example, Sorte, et al.^18^ estimated that SO_x_ and PM with particle size < 10 µm diameter (PM_10_) emissions from cargo ships could be reduced by 96% and 85% if 0.1%-sulfur instead of 2.7%-sulfur fuel is used, respectively. Wan et al.^19^ also estimated that SO_x_ and PM_10_ emissions from 28 vessels berthing at the Shanghai Port could be reduced by 81.2% and 71.1% if they switched to 0.1%-sulfur instead of 0.5%-sulfur fuel, respectively, while NO_x_ reduced only by 4.7%.

Shore power, which provides electric power to ships hoteling at berths, is ideal for eliminating ship emissions at ports^20^. Wan et al.^21^ concluded that shore power systems had the highest comprehensive emission reduction effect compared to switching to low-sulfur fuels and improving quay crane efficiency at a Shenzhen container terminal. Vaishnav, et al.^22^ estimated that air quality benefits of 70–150 million US dollars per year would be realized when a quarter to two-thirds of all ships calling at US ports used shore power. The estimated benefits accounted for the cost of installing shore power equipment at ports and retrofitting the ships with shore power connectors. Currently, Californian ports require ships to utilize shore power systems while at berth. Europe aims to have all the ports use shore power by 2025^23^. In Asia, several ports in China, the Port of Busan in South Korea, the Port of Osaka in Japan, and the Port of Kaohsiung in Taiwan also have shore power systems. Although shore power will improve air quality at the port, the source of the shore power should also be considered. Chang and Wang^24^ argued that shore power generated from coal-fired power plants with advanced air pollution control devices would still be better than not using shore power. For these reasons, shore power can be considered a green and sustainable best practice for ports.

At ports without shore power, ship generators, also known as gensets or auxiliary engines, provide energy for every ship's function, e.g., starting the main engine, powering loading/unloading equipment, refrigerated containers (reefers), central cooling water systems, and air conditioning. Generators are the primary power source when the ship is anchored or moored at a port. While the main engine is primarily used for the propulsion system while sailing, the generator remains responsible for all other power requirements even while sailing^25,26^.

Ship-related studies mostly use Automatic Identification System (AIS) data to estimate ship fuel consumption, engine power estimation^27^, and air pollution emissions (Chen et al. 2021), and not real-time sailing data, which would be more preferable for accurate estimations. Therefore, this study examines the real-time energy consumption and air pollutant emissions of PM_10_ and PM_2.5_, NO_x_, SO_x_, carbon monoxide (CO), and hydrocarbons (HC) of a container ship sailing from the West Coast of the US to East Asia through the Pacific Ocean. These air pollutants are of particular interest to air quality monitoring and control. Container ships are of interest because, despite the short stays at ports relative to other types of vessels, they contribute significantly to the emissions at ports^28^. The transpacific crossing is the busiest shipping route, with 25.1 million twenty-foot equivalent units (TEU) traded between East Asia and North America in 2020^29^. The container ship has a capacity of 8508 TEUs and is equipped with a generator engine (MAN 7L32/40). The vessel visited seven ports: Port of Los Angeles, Port of Oakland, and Port of Tacoma on the West Coast, and Port of Kaohsiung, Port of Hong Kong, Yantian International Container Terminals, and Port of Taipei in East Asia. In addition, the factors influencing energy consumption and the benefit of shore power were assessed. The results from this study would be useful for global and regional regulatory bodies in evaluating and improving the current regulations to ensure energy efficiency and reduce air pollutant emissions from container ships.

## Materials and methods

### Description of the container ship

The container ship was manufactured in 2013 and complies with the IMO Tier 2 global emission standards. It has a length of 334.8 m and a maximum draft of 14.2 m, with a deadweight of 105,000 tons and an 8508 twenty-foot equivalent unit (TEU) capacity. The reefer units are designed with a temperature of − 18 °C. The ship has a maximum speed of 24.5 knots, with a power output of 56,070 kW and a shaft speed of 97 rpm, and is equipped with a four-stroke medium-speed (720 rpm) generator engine (MAN 7L32/40) with a power output of 3200 kW. Detailed technical specifications of the ship are presented in Table S1. Shore power equipment was installed on both sides of the ship. There are no advanced marine emission control systems and marine exhaust treatment systems onboard.

### The transpacific voyage

**Figure 1:**
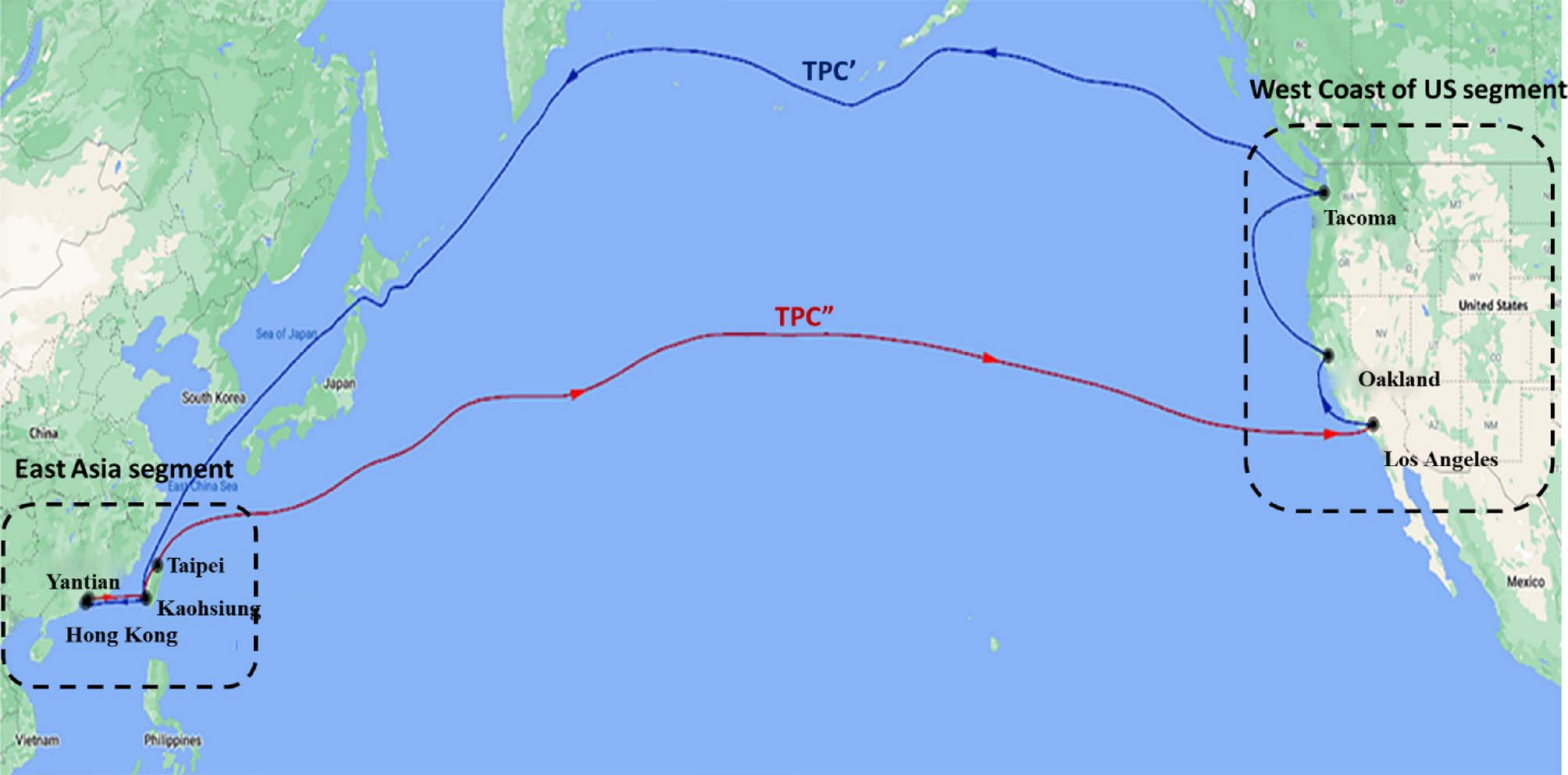
The round-trip voyage of the container ship starting from the Port of Los Angeles. Map retrieved from Google Maps (Map data @ 2022 Google). The blue line represents the transpacific crossing (TPC′) to the East Asian ports (EA), while the red line represents the transpacific crossing (TPC″) back to the West Coast (WC). The distances in WC = 1145 nmi; TPC′ = 6561 nmi; East Asia = 949 nmi; and TPC″ = 5949 nmi. (Figure prepared with Adobe Photoshop 21 - https://www.adobe.com/).

The two round trips of the container ship from the Port of Los Angeles (LAX) on the West Coast of the US to the East Asia regional ports, presented in Fig. 1, took 83 days. The first round trip voyage from the West Coast of the US to East Asia took 42 days (August 1st to September 11th, 2019), while the second round trip took 41 days (September 12th to October 22nd, 2019). In addition, the ship docked at six other ports: Port of Oakland (OKL), Port of Tacoma (TCM), Port of Kaohsiung (KHH), Port of Hong Kong (HKG), Yantian International Container terminals (YTT), and Port of Taipei (TPE) to offload and load containers.

One complete round-trip voyage was divided into four segments to explore the energy consumption and air emissions of the ship in different operating modes:

1. The West Coast of the US segment (WC) refers to the voyage in the regional ports on the West Coast of the US, starting from LAX → OKL → TCM with a total distance of 1145 nmi.
2. The Transpacific crossing (TPC′) refers to sailing from the Port of Tacoma to the Port of Kaohsiung in East Asia, through the Gulf of Alaska, Bering Strait, Tsugaru Strait, Sea of Japan, East China Sea, and Taiwan Strait, following the higher latitude crossing. The total distance of this segment was 6561 nmi.
3. The East Asia segment (EA) refers to the voyage in the East Asia regional ports, starting from KHH → HKG → YTT → KHH → TPE with a total distance of 949 nmi.
4. The Transpacific crossing (TPC″) refers to the transpacific crossing from East Asia to the West Coast of the US, starting from TPE to the LAX, following the lower latitude crossing. The total distance of this segment was 5949 nmi.

A “-1” or “-2” was added after a voyage segment to indicate the first or second round trips, e.g., EA-1 and EA-2 denote the first and second voyage in the East Asia regional ports, respectively. The average speed of the vessel was 20 knots on the open seas, and the speed of the engine was 73 rpm at about 42% of the maximum continuous rating. While entering a port area, the ship decelerated in accordance with each port authority’s requirements. As a result, the ship’s speed was lower than or equal to 12 knots before entering a port. The ship used heavy fuel oil (HFO, 2.7% m/m sulfur) in the open seas and ultra-low-sulfur fuel oil (0.1% m/m, ULSFO) 200 nautical miles near the West Coast of the US. In East Asia, the change from HFO to ULSFO was carried out at 20 nautical miles near the port area.

### Estimation of air pollutant emissions from the ship

To assess PM_10_, PM_2.5_, NO_X_, SO_x_, CO, and HC emissions from the ship power generators, the operational parameters, including wind, sea temperature, type and amount of fuel oil consumed, and energy consumption, were recorded during each voyage segment. Wind data was recorded via anemometers, and seawater temperature was recorded using a seawater thermometer. The total number of refrigerated containers onboard the ship was also carefully monitored and recorded. The availability and utilization of shore power electricity at the ports were also factored in the assessment. The energy of the entire ship, supplied by the generator (in kWh), was transmitted to the control dashboard through the SaCoS_one_ GenSet digital transmitter. The real-time data on energy generation was recorded every 3 s and stored as hourly average for each 12-h period. The recorded data were quality controlled to remove abnormal data and outliers. The energy consumption throughout the voyage is depicted in Fig. S1. Furthermore, air pollution emissions were based on the emission estimation methodology developed jointly by the California Air Resources Board (CARB), the South Coast Air Quality Management District (AQMD), the Port of Los Angeles, Long Beach Port, and the United States Environmental Protection Agency (USEPA)^30^.

1 $${E}_{i}=Energy \times {EF}_{i} \times {FCF}_{i} \times {CF}_{i}$$

where ${E}_{i}$ is the hourly air emission (g) from the ship’s generator for pollutant *i*; $Energy$ is the energy consumed by the ship (kWh); ${EF}_{i}$ is the emission factor of pollutant *i* (g kWh^−1^); ${FCF}_{i}$ is the fuel correction factor (dimensionless) for pollutant *i*; and ${CF}_{i}$ is the control factor of emission reduction technology (dimensionless) for pollutant *i*. Details of the emission factors at various conditions and fuel correction factors (FCF), which are adjusted according to the type of fuels used, and engine model year, are presented in Tables S2 and S3, respectively. The CF factors were set as one since no emission reduction devices were installed on the ship.

### Statistical analysis

Multiple linear regression (MLR) was used to investigate the relationship between the average energy consumption of the ship and influencing factors, including the total number of reefers, sea temperature, and wind. The MLR equation was expressed as follows:

2 $${\text{Y}} = \beta_{0} + \beta_{1} {\text{X}}_{1} + \beta_{2} {\text{X}}_{2} + \cdots + \beta_{m} {\text{X}}_{m}$$

where Y is the dependent variable, X_1_, X_2_,…,X_m_ are the independent variables, and ß_0_, ß_1_,…, ß_m_ are the regression coefficients. In this study, the average energy consumption was the dependent variable. Independent variables were determined based on the stepwise method. The stepwise approach was constructed recursively by adding or deleting one independent prediction at each time, and predictors that were not statistically significant were eliminated. In order to eliminate independent variables with multicollinearity, collinearity diagnostics among multiple independent variables were performed to ensure that the variance inflation factor (VIF) was < 10 or tolerance > 0.1. As a result, the total number of reefers, sea temperature, and wind (tailwind and headwind) were chosen as the independent variables. R^2^, adjusted R^2^, VIF, and the F ratio were used to analyze the relationship. In the statistical test, a 95% confidence interval was used as the decision index, and *P* < 0.05 was used as the criterion for statistical significance. The Cohen’s G-power statistical power analysis was used to estimate the appropriate sample size^31^.

## Results and discussion

### Energy consumption of the ship during the voyage

**Figure 2:**
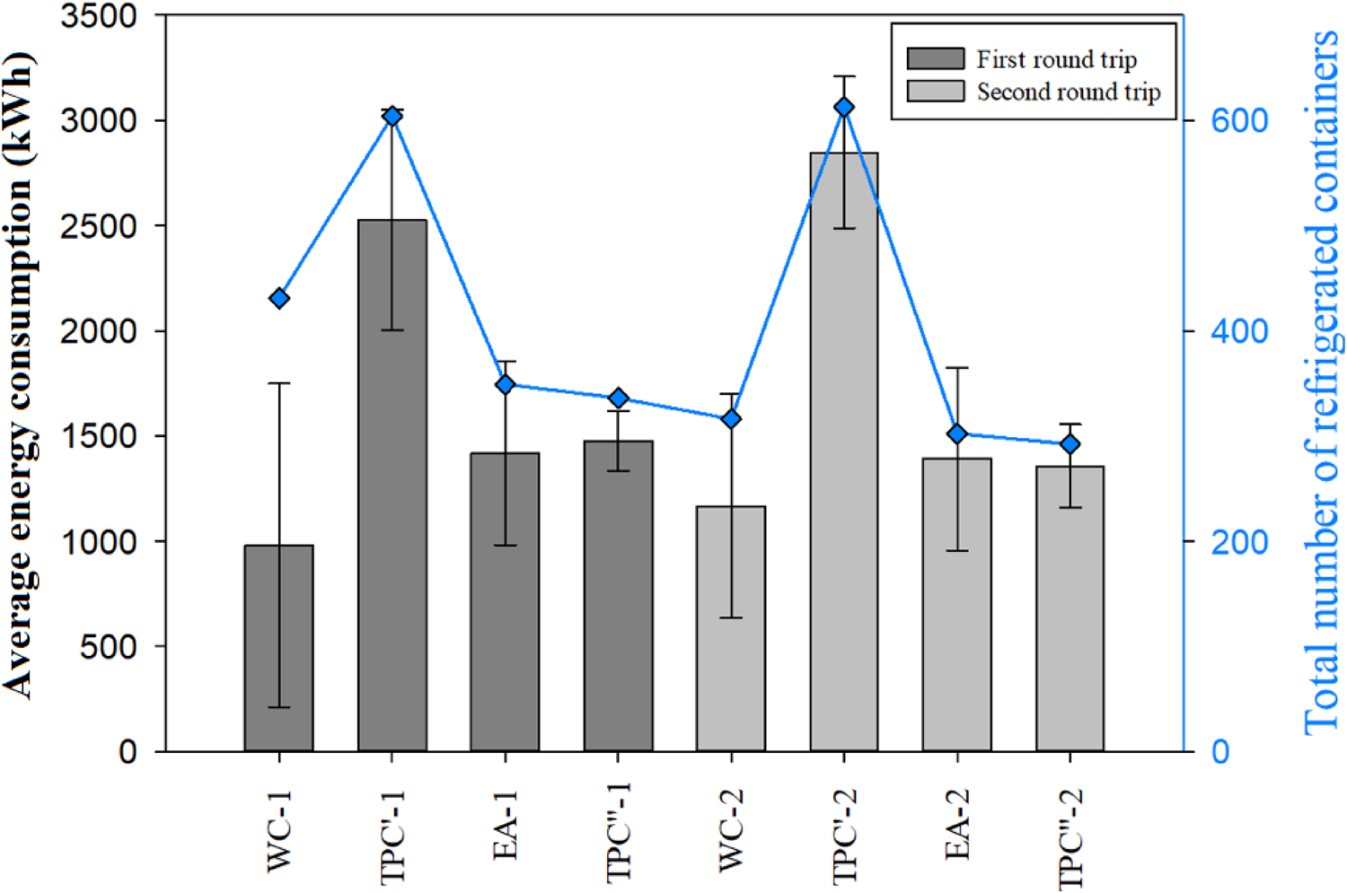
Average energy consumption and the number of refrigerated containers on board the vessel at the different voyage segments. *WC* west coast of the US, *TPC′* transpacific crossing to the East Asian ports, *EA* East Asia, *TPC″* transpacific crossing heading back to the Port of Los Angeles.

The average energy consumption of the ship during the first and second round trips is depicted in Fig. 2. The corresponding total energy consumption of the two round trips was 1.68 GWh and 1.75 GWh, respectively, as shown in Table 1. The energy consumption for the two round trips was statistically similar (Table S4).

**Table 1:** Total energy consumption and total air pollution emissions of each sailing segment.

	Sailing segment	Total energy consumption (MWh)	PM_10_ (kg)	PM_2.5_ (kg)	NO_X_ (kg)	SO_x_ (kg)	CO (kg)	HC (kg)
First round trip	WC-1	188	8	9	1864	3	264	113
	TPC′-1	728	1092	874	8154	8955	801	291
	EA 1	340	282	227	3603	2255	421	168
	TPC″-1	425	637	510	4757	5224	467	170
	Total	1681	2019	1620	18,378	16,437	1953	742
Second round trip	WC- 2	224	10	11	2216	4	313	134
	TPC′-2	854	1282	1025	9570	10,510	940	342
	EA-2	250	176	142	2626	1396	351	150
	TPC″-2	423	634	508	4738	5203	465	169
	Total	1751	2102	1686	19,150	17,113	2069	795

Generally, the transpacific crossing segments had the highest average energy consumption, followed by the EA segment, while the WC segment had the lowest average energy consumption, as shown in Fig. 2. For example, in the first round trip, the transpacific crossing segment to the East Asian ports (TPC′-1) had the highest average energy consumption of 2528 ± 523 kWh, while the West Coast segment (WC-1) had the lowest (980 ± 769 kWh).

Two of the three ports along the West Coast, namely, the Port of Los Angeles and the Port of Oakland in California, have shore power and strict regulations to ensure ships use shore power while at berth^32^. Therefore, the container ship only used the power generator at the Port of Tacoma and when sailing from one port to another. This explains the lower energy consumption during the WC segments. On the other hand, the container ship only used shore power at the Yantian International Container terminals during the East Asia segment. As a result, the average energy consumption of the EA segments was significantly higher than in the WC segments. For instance, the energy consumption during the EA segment was 30% higher than in the WC segment in the first round trip.

The average energy consumption during the two transpacific crossing segments, TPC′ and TPC″, were starkly different. For example, TPC′ was 41.7% higher than that of TPC″ in the first round trip and was 52.4% higher in the second round trip. The segments had different routes, as shown in Fig. 1. There were Beaufort scale 3–6 headwinds during the navigation through the TPC′ segment. In contrast, there were Beaufort scale 3–5 tailwinds during the navigation through the TPC″ segment.

Additionally, there were almost twice as many refrigerated containers onboard the ship during the TPC′ than the TPC″ voyage segment (604 vs. 336 TEU), as shown in Table S5. Refrigerated containers store and transport temperature-sensitive goods, including perishable food, pharmaceutical, and chemical products. Cariou et al.^33^ reported that reefers account for 10% of the total carrying capacity of container ships on a global average, and the energy consumption of the auxiliary engine is proportional to the number of reefers transported. Besides the wind and the number of reefers, the sea temperature also influences energy consumption. Seawater is usually pumped and used in the central cooling water system of the vessel to dissipate the heat and avoid malfunctioning or breakdown of machinery^34^. Aijjou et al.^34^ estimated that the central cooling pumps could account for as much as 10% of the total generator power consumed by the ship over time. Further analyses and discussions of these factors are presented in “Relationship between energy consumption and influencing factors”.

### Air emissions from the ship during the voyage

The emission rates of PM_10_, PM_2.5_, NO_x_, SO_x_, CO, and HC were directly proportional to the energy consumption of the ship. According to Eq. (1), TPC′ had the highest average emission rate, followed by TPC″, EA, and WC segment (Table S5), similar to the energy consumption trend. By multiplying the average emission rates at each segment by the cruising hours, the resulting average total air emission during the first and second round trips was 42.1 tons (Table 1).

**Figure 3:**
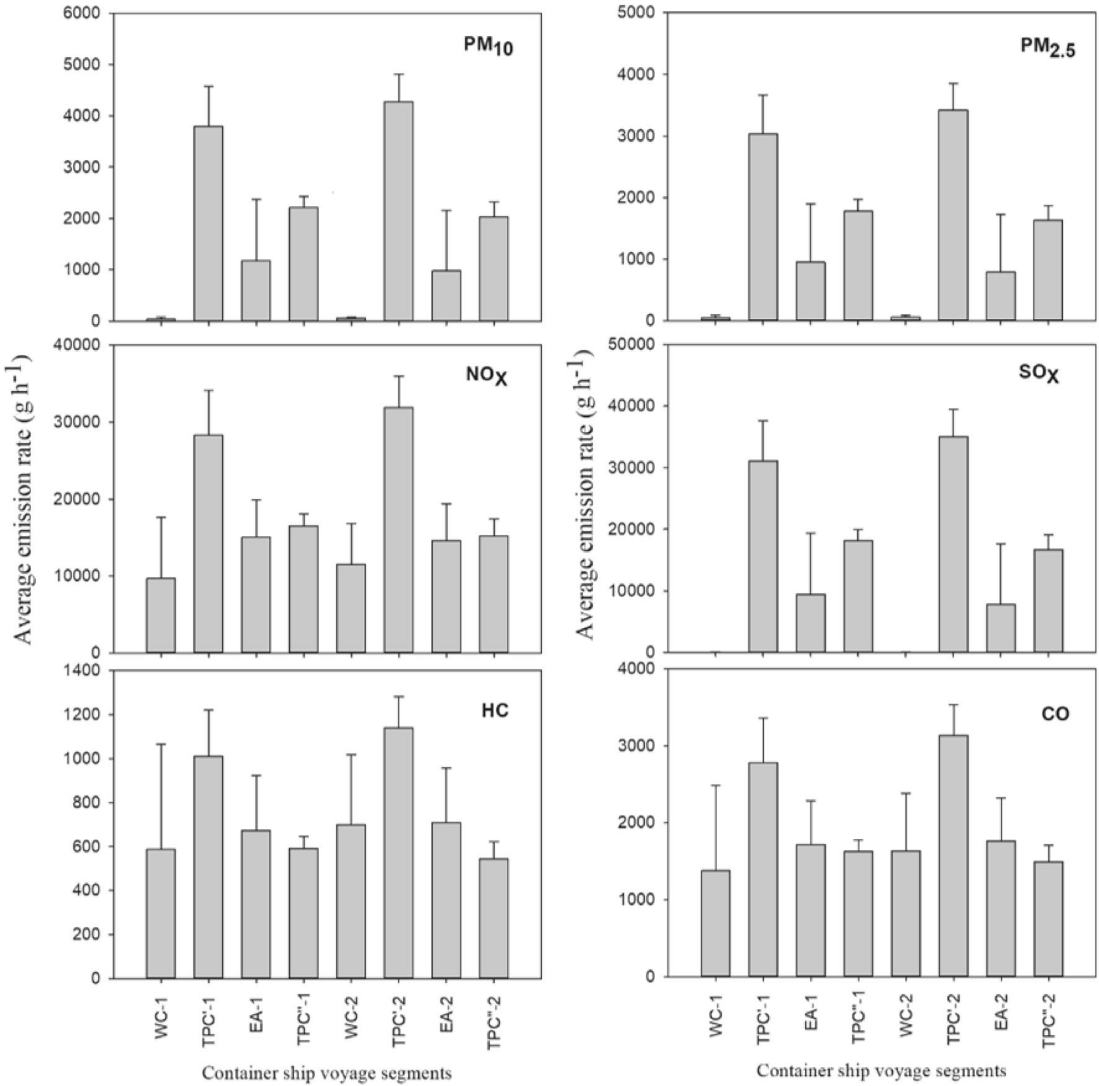
The estimated emission rates of the air pollutants from the container ship at the different voyage segments. *WC* west coast of the US, *TPC′* transpacific crossing to the East Asian ports, *EA* East Asia, *TPC″* transpacific crossing heading back to the Port of Los Angeles.

NO_x_ and SO_x_ were the main pollutants, followed by PM_10_, PM_2.5_, CO, and HC, as shown in Fig. 3. The NO_x_ and SO_x_ emissions were about 85% of the total emission quantities at each segment during the two round trips. Furthermore, most of the emissions were released during the transpacific crossing, followed by East Asia and at the West Coast of US segments, as shown in Fig. 3 and Fig. S2.

During the transpacific crossing, the navigational speeds were higher at around 20 knots, coinciding with high energy consumption and emissions. Also, the ship used HFO fuel with a higher sulfur content of 2.7%. As a result, the fraction of SO_x_ to the total emission was highest during the transpacific crossing segments, accounting for around 44%. On the other hand, for the WC segment, where 0.1% ULSFO was used, NO_x_ was the main pollutant, accounting for 82% of the total air emissions.

Emissions of these pollutants were influenced by fuel consumption of the ship (influenced by navigation speed and onshore power utilization) and fuel type. While approaching the ports, the ship slowed down as regulated by the port authorities^3^. For instance, the average speed decelerated to less than 12 knots while approaching the Port of Kaohsiung. Speed reduction has been historically used to reduce fuel consumption and is currently used to control ship emissions^35–37^.

### Benefits from switching HFO to ULSFO

**Figure 4:**
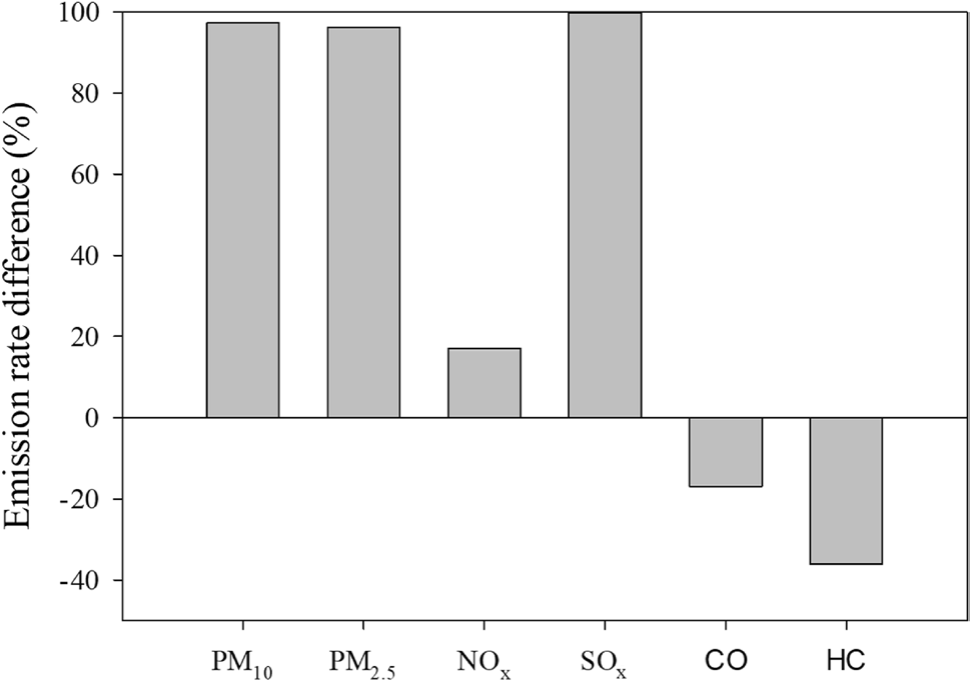
The emission rate differences of air pollutants from the ship while using ULSFO and HFO in the inter ports. ULSFO was used in the West Coast region while HFO was used in East Asia. Equation (3) was used for calculating the difference.

ULSFO instead of HFO has been shown to greatly reduce air pollutant emissions in our analysis. Equation (3) was used to estimate the emission rate (ER) differences for air pollutant *i* released by the ship within the inter ports in the East Asia segment, where HFO 2.7% m/m was used, and West Coast of the US segments, where ULSFO 0.1% m/m was used. The inter ports refer to the sailing of the ship from port to port within a segment. ${ER(EA)}_{i}$ and ${ER(WC)}_{i}$ represent the emission rate of pollutant *i* within the inter port area of East Asia and West Coast segment, respectively. The two segments had comparable average energy consumption at the inter ports, e.g., 1515 ± 115 kWh and 1526 ± 333 kWh for the WC-1 and EA-1, respectively (Table 2). The emission rate differences are shown in Fig. 4.

**Table 2:** Average energy consumption and emission of the six air pollutants from the auxiliary engine while inter ports within the West Coast and East Asia segments.

Inter ports within segments	Average energy consumption (kWh)	Fuel type	Average estimated emission (g h^−1^)					
			PM_10_	PM_2.5_	NO_X_	SO_x_	CO	HC
WC-1	1515 ± 115	ULSFO	65 ± 5	72 ± 5	14,995 ± 1141	25 ± 1	2121 ± 161	909 ± 69
EA-1	1526 ± 333	HFO	2289 ± 499	1831 ± 399	17,091 ± 3732	18,769 ± 4098	1678 ± 366	610 ± 133
WC-2	1437 ± 221	ULSFO	62 ± 7	69 ± 8	14,231 ± 1670	24 ± 2	2012 ± 236	862 ± 101
EA-2	1573 ± 173	HFO	2360 ± 260	1888 ± 208	17,623 ± 1942	19,354 ± 2133	1730 ± 190	629 ± 69
WC	1476 ± 144	ULSFO	64 ± 6	71 ± 7	14,614 ± 1422	25 ± 2	2067 ± 201	996 ± 86
EA	1544 ± 278	HFO	2613 ± 417	1853 ± 333	17,291 ± 3112	18,989 ± 3418	1698 ± 306	618 ± 111

3 $$\Delta ER \left(\mathrm{\%}\right)=\frac{ER(EA)- ER(WC)}{ER(EA)}$$

The average energy consumption and emission rates of the six air pollutants released while using HFO and ULSFO are presented in Table 2. PM_10_, PM_2.5_, and SO_x_ decreased by > 96%, while NO_x_ decreased by only 17.0%. The results are in good agreement with Sorte et al.^18^ and Browning et al.^38^, that reported SO_x_ could be reduced by up to 96% and 99% after switching to 0.1%-sulfur fuel ULSFO.

On the contrary, CO and HC increased by 16.9% and 36.1%, respectively. CO and HC are products of incomplete combustion, and the increase of these pollutants suggests that incomplete combustion of the fuel occurs when the fuels are switched. Therefore, although switching to ULSFO has its merits, it could increase the emission of products of incomplete combustion and fuel consumption. Therefore, the generator engine combustion efficiency needs to be optimized for ULSFO, which warrants further research.

### Relationship between energy consumption and influencing factors

The energy consumption of ships is not only affected by the total number of refrigerated containers but also the environmental factors when sailing. Therefore, multiple linear regression (MLR) analysis was used to understand the influence of these factors on the energy consumption of the ship on this route. The data for the two round trips, with a sample size of 139, were used in the MLR analysis. This met the appropriate sample size (≥ 77) calculate by the Cohen’s G-power statistical power analysis.

The key influencing factors, determined by the stepwise method, were the total number of reefers, tailwind and headwind, and seawater temperature. The three factors (total number of reefers, tailwind and headwind, and seawater temperature) could explain 82.9% of the variation in energy consumption (Table S6). The tolerance values were > 0.1, and VIF values were < 10, revealing that MLR models had no collinearity problem. Equation (4) shows the relationship between energy consumption and the three influencing factors.

4 $$\begin{aligned} {\text{Ave}}{.}\,\,{\text{power}}\,\,{\text{consumption}}\,\,{\text{(kWh)}} & = - { 389}.{81 } + { 4}.{38} \times {\text{total}}\,\,{\text{number}}\,\,{\text{of}}\,\,{\text{reefers}} \\ & \quad + { 2}0.{84 } \times {\text{ sea temperature }} + { 148}.{38 } \times {\text{Tailwind or Headwind}} \\ \end{aligned}$$

The Pearson correlation coefficient showed the total number of refrigerated containers had a strong positive correlation with the energy consumption of the ship’s generators (r = 0.876) at *P* < 0.001, as shown in Table 3. On the other hand, the tailwind and headwind had a weak positive correlation with the energy consumption (r = 0.307), and no significant correlation was found between sea temperature and energy consumption (r = 0.035) at *P* < 0.001. Therefore, the total number of reefers was the main factor influencing energy consumption.

**Table 3:** Statistical analysis between energy consumption and the influencing factors.

Items	Energy consumption	Number of refrigerated containers loaded	Sea temperature	Tailwind and headwind
Energy consumption	1.000	0.876***	0.035	0.307***
Number of refrigerated containers loaded	0.876***	1.000	− 0.209**	0.191
Sea temperature	0.035	− 0.209**	1.000	0.125
Tailwind and headwind	0.307***	0.191	0.125	1.000

*P < 0.05, **P < 0.01, ***P < 0.001.

If we ignore wind and sea temperature, the energy consumption will increase by 4.38 kWh for each additional reefer loaded onboard the ship. Taking the maximum of 8508 TEU for the container ship, the total number of refrigerated containers that can be carried is 948 TEU. Therefore, when sailing on the open seas, the hourly energy consumption would be 4.15 MWh, and the corresponding air emission rate would be 117 kg h^−1^ (according to Eq. 1). Therefore, the emissions would amount to 36.5 tons of the six pollutants emitted during the transpacific crossing. Similarly, the hourly energy consumption for a modern standard container ship with about 24,000 TEU, of which about 2600 TEU would amount to 11.4 MWh. The estimated air emission rate would be 321 kg h^−1^, amounting to 100 tons of the six pollutants emitted during the transpacific crossing (TPC′-1) segment. These results imply that reducing the energy consumption of reefers would greatly reduce the emissions from container ships. This can be done by improving the energy efficiency of the reefers. In addition, using cleaner energy sources, especially in port areas, should be the ultimate goal of port authorities and the various stakeholders.

### Benefits of adopting shore power

Five of the seven ports visited, namely, LAX, OKL, TCM on the West Coast, and KHH and YTT in the East Asia region, have installed shore power systems. However, the ship was connected only to the shore power system in LAX, OKL, and YTT. Therefore, the ship’s generator engine was continuously operated to meet the power requirements of the ship while it was moored at the other ports. Table S7 shows the average energy consumption and the emission rate of the pollutants from the ship at the four ports where shore power was not used. The average energy consumption at the four ports and emission rates was 1516 ± 249 kWh and 18.10 ± 3.08 kg h^−1^, respectively. The total hours spent at the berths of these four ports was 156 h, and therefore, the average total air emission from the ship at these ports was around 2.88 tons. These emissions from the container ship would be prevented through shore power adoption, resulting in zero air pollutant emissions from the ship. Instead, the emissions will be reflected in the shore power source and in the power transmission. Because power plants are centralized and stationary, their emissions are relatively easier to regulate and control. Nonetheless, renewable sources are recommended to meet sustainable goals. Therefore, shore power systems can improve the air quality of the port and the surrounding environment, especially since most of these ports are in densely populated cities with millions of residents^4,24^.

### Limitations and uncertainties

This study followed the emission estimation methodology from the Port of Los Angeles (POLA)^30^. The method resulted from the joint research of the California Air Resources Board (CARB), the South Coast Air Quality Management District (AQMD), the Port of Los Angeles, Long Beach Port, and the United States Environmental Protection Agency (USEPA). It is the most common method for estimating emissions from sources. However, they do not offer information regarding the representativeness and accuracy of the selected emission factors in their report. The selected emission factor is based on the fuel consumed and the engine's age and type, as shown in Table S2. Some factors that might influence the accuracy of the selected emission factors include maintenance practices which would lead to variations in the efficiency and emissions of an engine, and ambient conditions, which would influence the emissions of certain pollutants^39^. Furthermore, although the explanatory power of the MLR model reached 82.9%, the lack of actual data to verify the relationship between the ship’s energy consumption and the three variables limits the study.

## Conclusions

This study investigated the real-time energy consumption and air pollutant (PM_10_, PM_2.5_, NO_x_, SO_x_, CO, and HC) emissions from a container ship’s generator during transpacific voyages. The transpacific crossing segments in the high seas had the highest energy consumption (2528 ± 523 kWh) and air emissions (70 kg h^−1^). The two Californian ports (Port of Los Angeles and Oakland) have strict emission regulations requiring ships to switch to ultra-low-sulfur fuel oil (< 0.1% sulfur m/m) near the port and to use shore power while docking at the port. As a result, the West Coast of the US segment had the lowest energy consumption and air emissions. Although switching to low-sulfur fuel significantly reduces PM, SO_x_, and some NO_x_ emissions, the emissions of CO and HC increased by 16.9% and 36.1%, respectively. The increase of these products of incomplete combustion signals that the combustion efficiency is compromised while using low-sulfur fuel. As a result, more fuel consumption is expected with the adoption of low-sulfur fuel. The energy consumption was primarily affected by the total number of reefers onboard (r = 0.876 at *P* < 0.001). Therefore, the energy efficiency optimization of reefers is a key research area in reducing energy consumption and related air emissions. The relationship between the ship’s energy consumption and the three variables can be verified using different ships. Additionally, actual measurements of emissions of air pollutants, including CO_2_, VOC, and persistent organic pollutants, should be carried out. Shore power adoption seems to be a better option for the ships at berth. However, this would be costly for the port authorities and ship owners and require significant government incentives and cooperation among stakeholders. Moreover, the source of the shore power needs to be carefully considered. The use of nonrenewable sources such as traditional coal-fired power plants would only transfer the emissions to a different location. On the other hand, renewable energy sources, including solar, wind, and geothermal, would ensure shore power systems are truly green and sustainable.

## Supplementary Information

Supplementary Information.

## Data Availability

The datasets used and/or analysed during the current study available from the corresponding author on reasonable request.

## References

[1] UNCTAD. Review of maritime transport 2018. New York (2018).

[2] Chen D (2020). The impact of ship emissions on nitrogen and sulfur deposition in China. Sci. Total Environ..

[3] Sadiq, M. *et al.* Future greener seaports: A review of new infrastructure, challenges, and energy efficiency measures. *IEEE Access* (2021).

[4] Nguyen, D. H. *et al.* Reduction of NOx and SO2 emissions by shore power adoption. *Aerosol. Air Qual. Res.* **21**. 10.4209/aaqr.210100 (2021).

[5] Ammar NR, Seddiek IS (2020). An environmental and economic analysis of emission reduction strategies for container ships with emphasis on the improved energy efficiency indexes. Environ. Sci. Pollut. Res. Int..

[6] Mocerino L, Murena F, Quaranta F, Toscano D (2020). A methodology for the design of an effective air quality monitoring network in port areas. Sci. Rep..

[7] Wang S, Notteboom T (2015). The role of port authorities in the development of LNG bunkering facilities in North European ports. WMU J. Marit. Aff..

[8] Zhong Q (2020). Global sulfur dioxide emissions and the driving forces. Environ. Sci. Technol..

[9] Lee HH, Iraqui O, Wang C (2019). The impact of future fuel consumption on regional air quality in Southeast Asia. Sci. Rep..

[10] IMO. Third IMO Greenhouse Gas Study 2014 Executive Summary and Final Report. . (2015).

[11] Gössling, S., Meyer-Habighorst, C. & Humpe, A. A global review of marine air pollution policies, their scope and effectiveness. *Ocean Coast. Manag.* **212**. 10.1016/j.ocecoaman.2021.105824 (2021).

[12] Van TC, Ramirez J, Rainey T, Ristovski Z, Brown RJ (2019). Global impacts of recent IMO regulations on marine fuel oil refining processes and ship emissions. Transp. Res. D Transp. Environ..

[13] Ammar NR, Seddiek IS (2021). Evaluation of the environmental and economic impacts of electric propulsion systems onboard ships: Case study passenger vessel. Environ. Sci. Pollut. Res. Int..

[14] International Maritime Organization, I. *IMO sets 2020 date for ships to comply with low sulphur fuel oil requirement*, http://www.imo.org/en/MediaCentre/PressBriefings/Pages/MEPC-70-2020sulphur.aspx (2016).

[15] Wang S, Notteboom T (2014). The adoption of liquefied natural gas as a ship fuel: A systematic review of perspectives and challenges. Transp. Rev..

[16] International Maritime Organization, I. *IMO 2020—cutting sulphur oxide emissions*, http://www.imo.org/en/MediaCentre/HotTopics/Pages/Sulphur-2020.aspx (2020).

[17] CARB. Notification of requirements to owners and operators of ocean going vessels for complying with the california ocean going vessel fuel regulations. California Air Resource Board (2020).

[18] Sorte S, Rodrigues V, Lourenço R, Borrego C, Monteiro A (2021). Emission inventory for harbour-related activities: Comparison of two distinct bottom-up methodologies. Air Qual. Atmos. Health.

[19] Wan Z, Zhang Q, Xu Z, Chen J, Wang Q (2019). Impact of emission control areas on atmospheric pollutant emissions from major ocean-going ships entering the Shanghai Port China. Mar. Pollut. Bull..

[20] Wang S, Qi J, Laporte G (2022). Optimal subsidy design for shore power usage in ship berthing operations. Nav. Res. Logist..

[21] Wan Z (2021). Evaluation of emission reduction strategies for berthing containerships: A case study of the Shekou Container Terminal. J. Clean. Prod..

[22] Vaishnav P, Fischbeck PS, Morgan MG, Corbett JJ (2016). Shore power for vessels calling at US ports: Benefits and costs. Environ. Sci. Technol..

[23] Zis TP (2019). Prospects of cold ironing as an emissions reduction option. Transp. Res. Part A Policy Pract..

[24] Chang CC, Wang CM (2012). Evaluating the effects of green port policy: Case study of Kaohsiung harbor in Taiwan. Transp. Res. D Transp. Environ..

[25] Molland, A. F. *The maritime engineering reference book: a guide to ship design, construction and operation*. Elsevier, (2011).

[26] Czermański E, Cirella GT, Oniszczuk-Jastrząbek A, Pawłowska B, Notteboom T (2021). An energy consumption approach to estimate air emission reductions in container shipping. Energies.

[27] Kim S-H, Roh M-I, Oh M-J, Park S-W, Kim I-I (2020). Estimation of ship operational efficiency from AIS data using big data technology. Int. J. Nav. Archit. Ocean Eng..

[28] Merk, O. Shipping emissions in ports. *International Transport Forum* **20**, 1. 10.1787/5jrw1ktc83r1-en (2014).

[29] UNCTAD. Review of Maritime Transport. United Nations, New York, United States of America (2020).

[30] POLA. San Pedro Bay Ports Emissions Inventory Methodology Report. Los Angeles, CA, (2019).

[31] Cohen, J. *Statistical power analysis for the behavioral sciences*. 2nd ed, Routledge, (1988).

[32] Peng Y (2019). A method for determining the allocation strategy of on-shore power supply from a green container terminal perspective. Ocean Coast Manag..

[33] Cariou P, Parola F, Notteboom T (2019). Towards low carbon global supply chains: A multi-trade analysis of CO2 emission reductions in container shipping. Int. J. Prod. Econ..

[34] Aijjou, A., Bahatti, L. & Raihani, A. in *2018 4th International Conference on Optimization and Applications (ICOA).* 1–6 (IEEE).

[35] Zis T, North RJ, Angeloudis P, Ochieng WY, Bell MGH (2014). Evaluation of cold ironing and speed reduction policies to reduce ship emissions near and at ports. Marit. Econ. Logist..

[36] Zhen L, Wu Y, Wang S, Laporte G (2020). Green technology adoption for fleet deployment in a shipping network. Transp. Res. Part B Methodol..

[37] Qi J, Wang S, Psaraftis H (2021). Bi-level optimization model applications in managing air emissions from ships: A review. Commun. Transp. Res..

[38] Browning L, Hartley S, Bandemehr A, Gathright K, Miller W (2012). Demonstration of fuel switching on oceangoing vessels in the Gulf of Mexico. J. Air Waste Manag. Assoc..

[39] Frey, H. C. Quantification of uncertainty in emission factors and inventories. *Unpublished manuscript* (2007).

