# Supplementary material for: Real-time energy consumption and air pollution emission during the transpacific crossing of a container ship
Source: Sci Rep. 2022 Sep 10;12:15272. doi: 10.1038/s41598-022-19605-7 (PMC9464251; doi:10.1038/s41598-022-19605-7)
Supplement: Supplementary file 1 — Supplementary Information. [file 41598_2022_19605_MOESM1_ESM.docx]

Supplementary file for

**Real-time energy consumption and air pollution emission during the transpacific crossing of a container ship**

Chin-Ko Yeh^1^, Chitsan Lin^1,2*^, Hsueh-Chen Shen^1^, Nicholas Kiprotich Cheruiyot^3,4^, Duy-Hieu Nguyen^1^, Chi-Chung Chang^5^

^1^ Ph.D. Program in Maritime Science and Technology, National Kaohsiung University of Science and Technology, Kaohsiung 81157, Taiwan (R.O.C.)

^2^ Department of Marine Environmental Engineering, National Kaohsiung University of Science and Technology, Kaohsiung 81157, Taiwan (R.O.C.)

^3^ Super Micro Mass Research and Technology Center, Cheng Shiu University, Kaohsiung 833301, Taiwan (R.O.C.)

^4^ Center for Environmental Toxin and Emerging-Contaminant Research, Cheng Shiu University, Kaohsiung 833301, Taiwan (R.O.C.)

^5^ Department of Construction Management, College of Technology, University of Houston, Houston, Texas 77004, United States

*Corresponding author: Tel/Fax: +886-7-3651472; E-mail: ctlin@nkust.edu.tw


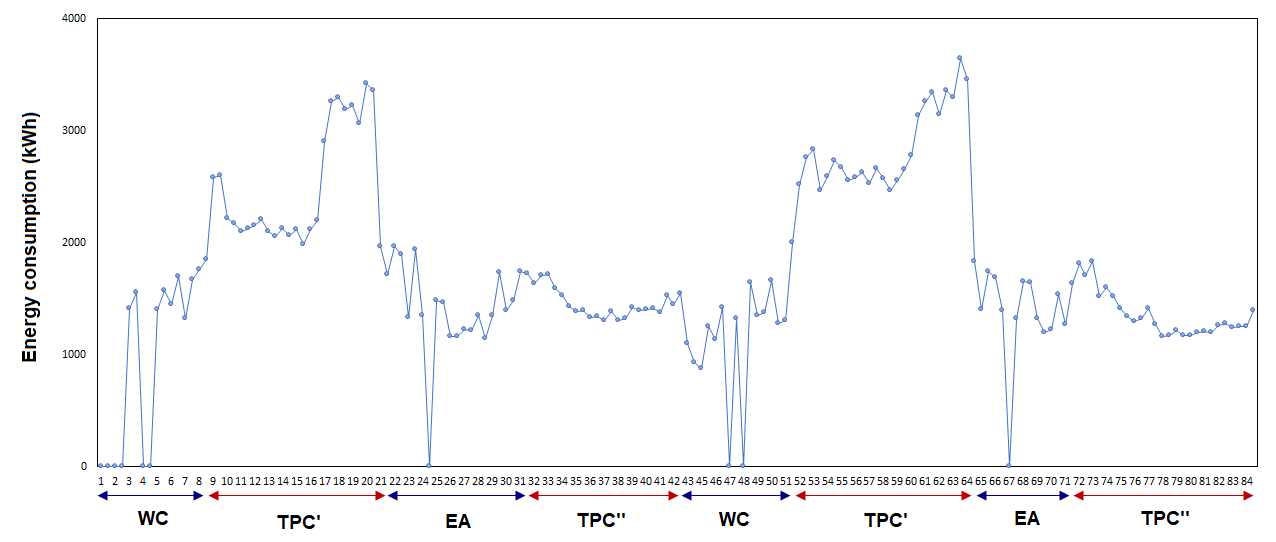


Figure S1. The container ship's power consumption (kWh) during the two round-trip voyages from the West Coast of the US to East Asia.


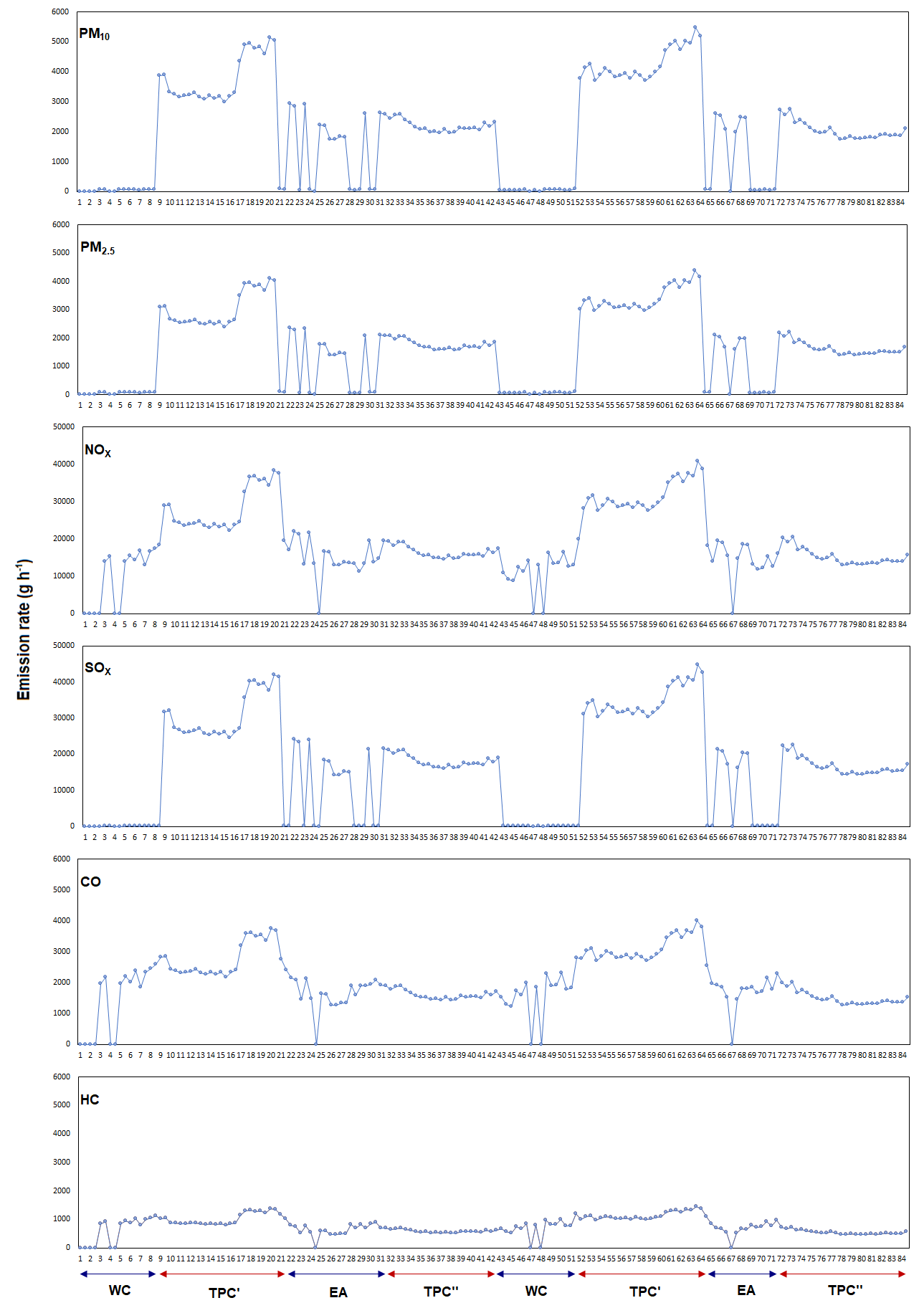


Figure S2. The container ship's emission rate (g h^-1^) during the two round-trip voyages from the West Coast of the US to East Asia.

Table S1. Technical specifications of the ship.

| Auxiliary engine | | |
| --- | --- | --- |
| No. of sets | | 4 sets |
| Prime mover | Type | 4 stroke turbo-charged, single action, trunk piston, fresh water cooled marine diesel engine |
|  | Model no. | Doosan-MAN 7L32/40 |
|  | kW x rpm | 3,500 x 720 |
| Generator | Type | Synchronous brushless totally enclosed water-cooled bracket type with forced-lubrication single bearing |
|  | Maker | NISHISHIBA |
|  | Output capacity x Voltage x Hz | 3200 kW x 6600 V x 60 Hz |
| Total weight of Genset (kg) | | 79000 |
| Maker | | Doosan Engine Co., Ltd. |

Table S2. Pollutant emission factors for auxiliary engines, g kWh^-1^

| Engine Category | IMO Tier | Model year | PM_10_ | PM_2.5_ | NO_x_ | SO_x_ | CO | HC |
| --- | --- | --- | --- | --- | --- | --- | --- | --- |
| Using 2.7%-Sulfur Heavy Fuel Oil (HFO) Fuel | | | | | | | | |
| Medium speed auxiliary | 0 | ≤1999 | 1.5 | 1.2 | 14.7 | 12.3 | 1.1 | 0.4 |
|  | I | 2000~2011 | 1.5 | 1.2 | 13 | 12.3 | 1.1 | 0.4 |
|  | II | 2011~2016 | 1.5 | 1.2 | 11.2 | 12.3 | 1.1 | 0.4 |
|  | III | ≥2016 | 1.5 | 1.2 | 2.8 | 12.3 | 1.1 | 0.4 |
| High speed auxiliary | 0 | ≤1999 | 1.5 | 1.2 | 11.6 | 12.3 | 0.9 | 0.4 |
|  | I | 2000~2011 | 1.5 | 1.2 | 10.4 | 12.3 | 0.9 | 0.4 |
|  | II | 2011~2016 | 1.5 | 1.2 | 8.2 | 12.3 | 0.9 | 0.4 |
|  | III | ≥2016 | 1.5 | 1.2 | 2.1 | 12.3 | 0.9 | 0.4 |
| Using 0.1%- Ultra low sulfur fuel oil (ULSFO) | | | | | | | | |
| Medium speed auxiliary | 0 | ≤1999 | 0.255 | 0.24 | 13.82 | 0.455 | 1.4 | 0.6 |
|  | I | 2000~2011 | 0.255 | 0.24 | 12.22 | 0.455 | 1.4 | 0.6 |
|  | II | 2011~2016 | 0.255 | 0.24 | 10.53 | 0.455 | 1.4 | 0.6 |
|  | III | ≥2016 | 0.255 | 0.24 | 2.63 | 0.455 | 1.4 | 0.6 |
| High speed auxiliary | 0 | ≤1999 | 0.255 | 0.24 | 10.9 | 0.455 | 1.1 | 0.5 |
|  | I | 2000~2011 | 0.255 | 0.24 | 9.78 | 0.455 | 1.1 | 0.5 |
|  | II | 2011~2016 | 0.255 | 0.24 | 7.71 | 0.455 | 1.1 | 0.5 |
|  | III | ≥2016 | 0.255 | 0.24 | 1.97 | 0.455 | 1.1 | 0.5 |

Source: San Pedro Bay Ports Emissions Inventory Methodology (POLA, 2019).

Note: IMO: international maritime organization

Table S3. Fuel Correction Factors for Ocean Going Vessels.

| Baseline Fuel (S%) | Fuel used (S%) | PM_10_ | PM_2.5_ | NOx | SO_x_ | CO | HC |
| --- | --- | --- | --- | --- | --- | --- | --- |
| HFO (2.7%) | HFO (2.7%) | 1 | 1 | 1 | 1 | 1 | 1 |
| HFO (2.7%) | VLSFO (0.5%) | 0.25 | 0.29 | 0.94 | 0.185 | 1 | 1 |
| HFO (2.7%) | ULSFO (0.1%) | 0.17 | 0.2 | 0.94 | 0.037 | 1 | 1 |

Sources: San Pedro Bay Ports Emissions Inventory Methodology Report (POLA, 2019)

Table S4. Comparison of the average power consumption and total air emissions

| Variables | Kruskal-Wallis test | Statistical inspection | Standard error | Standard verification statistics | Significance  (P value) | Significance after adjustment (P value) |
| --- | --- | --- | --- | --- | --- | --- |
| Power consumption (kWh) | WC-2 & WC-1 | 11.188 | 18.826 | 0.594 | 0.552 | 1.000 |
|  | TPC′-1 & TPC′-2 | -10.291 | 15.217 | -0.676 | 0.499 | 1.000 |
|  | EA-1 & EA-2 | -1.683 | 18.188 | -0.093 | 0.926 | 1.000 |
|  | TPC″-2 & TPC″-1 | 25.034 | 15.073 | 1.661 | 0.097 | 1.000 |
| Total air emissions (g h^-1^) | WC-2 & WC-1 | 3.250 | 18.827 | 0.173 | 0.863 | 1.000 |
|  | TPC′-1 & TPC′-2 | -10.249 | 15.218 | -0.674 | 0.501 | 1.000 |
|  | EA-2 & EA-1 | 3.683 | 18.189 | 0.203 | 0.840 | 1.000 |
|  | TPC″-2 & TPC″-1 | 14.263 | 15.074 | 0.946 | 0.344 | 1.000 |

Table S5. Shore power, hourly average power consumption, total number of refrigerated containers, and air emissions for each sailing segment.

| Sailing segments (days) | Port | Fuel oil | latitude (highest) | Shore power equip-ment | Shore power use | Generator’s Ave. power consumption  (kWh)  (M ± SD) | Total number of loaded refrigerated containers (TEU)  (M ± SD) | PM_10_ (g h^-1^)  (M ± SD) | PM_2.5_ (g h^-1^)  (M ± SD) | NO_X_ (g h^-1^)  (M ± SD) | SO_x_ (g h^-1^)  (M ± SD) | CO (g h^-1^)  (M ± SD) | HC (g h^-1^)  (M ± SD) |
| --- | --- | --- | --- | --- | --- | --- | --- | --- | --- | --- | --- | --- | --- |
| **WC-1 (1st~8th)** |  |  |  | 100% | 67% | 980 ± 796 | 431 ± 38 | 43 ± 34 | 47 ±38 | 9,710 ± 7,882 | 17 ± 13 | 1,373 ± 1,114 | 588 ± 477 |
|  | LAX | ULSFO | 33.736 | Yes | Yes | 0 | 420 ± 40 | 0 | 0 | 0 | 0 | 0 | 0 |
|  | OKL | ULSFO | 33.472 | Yes | Yes | 0 | 397 ± 18 | 0 | 0 | 0 | 0 | 0 | 0 |
|  | TCM | ULSFO | 47.254 | Yes | No | 1,651 ± 233 | 467 ± 49 | 71 ± 10 | 79 ± 11 | 16,344 ± 2,301 | 28 ± 4 | 2,312 ± 326 | 990±140 |
|  | Inter Ports | ULSFO |  |  |  | 1,515 ± 115 | 426 ± 12 | 65 ± 5 | 72 ± 5 | 14,995 ± 1,141 | 25 ± 1 | 2,121 ± 161 | 909±69 |
| **TPC′-1 (9th~21st)** |  | HFO | 48.518 |  |  | 2,528 ± 523 | 604 ± 0 | 3,792 ± 785 | 3,034 ± 628 | 28,313 ± 5,858 | 31,093 ± 6,434 | 2,781 ± 575 | 1,011 ± 209 |
| **EA-1 (22nd~31st)** |  |  |  | 50% | 25% | 1,418 ± 436 | 349 ± 38 | 1,174 ± 1,197 | 947 ±948 | 15,031 ± 4,835 | 9,395 ± 10,022 | 1,716 ± 567 | 672 ± 250 |
|  | KSG | ULSFO | 22.608 | Yes | No | 1,508 ± 329 | 359 ± 56 | 65 ± 14 | 72 ± 16 | 14,928 ± 3,258 | 25 ± 6 | 2,111 ± 461 | 905±198 |
|  | HKG | ULSFO | 22.324 | No | No | 1,340 ± 13 | 322 ± 0 | 58 ± 0.6 | 64 ± 0.6 | 13,259 ± 133 | 23 ± 0.2 | 1,473 ± 15 | 536±5 |
|  | YYT | ULSFO | 23.350 | Yes | Yes | 0 | 342 ± 0 | 0 | 0 | 0 | 0 | 0 | 0 |
|  | TPE | ULSFO | 25.161 | No | No | 1,443 ± 64 | 308 ± 3 | 62 ± 3 | 69 ± 3 | 14,278 ± 637 | 24±1 | 2,020 ± 90 | 866 ± 39 |
|  | Inter Ports | HFO |  |  |  | 1,526 ± 333 | 357 ± 31 | 2,289 ± 499 | 1,831 ± 400 | 17,091 ± 3,732 | 18,770 ± 4,099 | 1,679±367 | 610±133 |
| **TPC″-1 (32nd~42nd)** |  | HFO | 45.375 |  |  | 1,475 ± 143 | 336 ± 0 | 2,212 ± 215 | 1,785 ± 181 | 16,518 ± 1,601 | 18,140 ± 1,759 | 1,622 ± 157 | 590 ± 57 |
| **WC-2 (43rd~50th)** |  |  |  | 100% | 33% | 1,166 ± 532 | 316 ± 63 | 51 ± 23 | 56 ± 26 | 11,540 ± 5,265 | 20 ± 9 | 1,632 ± 745 | 700 ± 319 |
|  | LAX | ULSFO | 33.736 | Yes | No | 1,119 ± 201 | 296 ± 74 | 49 ± 9 | 54 ± 10 | 11,078 ± 1,990 | 19 ± 3 | 1,567 ± 281 | 672 ± 121 |
|  | OKL | ULSFO | 33.472 | Yes | Yes | 0 | 304 ± 19 | 0 | 0 | 0 | 0 | 0 | 0 |
|  | TCM | ULSFO | 47.254 | Yes | No | 1,656 ± 495 | 394 ± 131 | 72 ± 21 | 79 ± 24 | 16,391 ± 4,899 | 28 ± 8 | 2,318 ± 693 | 994 ± 297 |
|  | Inter Ports | ULSFO |  |  |  | 1,438  ± 169 | 313 ± 11 | 62 ± 7 | 69 ± 8 | 14,231 ± 1,671 | 24 ± 3 | 2,013 ± 236 | 862 ± 101 |
| **TPC′-2 (51st~63rd)** |  | HFO | 48.518 |  |  | 2,848 ± 361 | 613 ± 0 | 4,273 ± 541 | 3,418 ± 433 | 31,902± 4,042 | 35,035 ± 4,439 | 3,133 ± 397 | 1,139 ± 144 |
| **EA-2 (64th~71st)** |  |  |  | 100% | 50% | 1,391 ± 434 | 302 ± 44 | 977 ± 1,179 | 792 ± 935 | 14,592 ± 4,797 | 7,754 ± 9,886 | 1,759 ± 562 | 709 ± 248 |
|  | KSG | ULSFO | 22.608 | Yes | No | 1,429 ± 221 | 290 ± 58 | 62 ± 10 | 69 ± 11 | 14,142 ± 2,189 | 24 ± 4 | 2,000 ± 310 | 857 ± 133 |
|  | YYT | ULSFO | 23.350 | Yes | Yes | 0 | 294 ± 0 | 0 | 0 | 0 | 0 | 0 | 0 |
|  | Inter Ports | HFO |  |  |  | 1,574 ± 173 | 319 ± 10 | 2,360 ± 260 | 1,888 ± 208 | 17,623 ± 1,943 | 19,354 ± 2,133 | 1,731 ± 191 | 629 ± 69 |
| **TPC″-2 (72nd~83rd)** |  | HFO | 45.375 |  |  | 1,356 ± 197 | 292 ± 0 | 2,034 ± 295 | 1,627 ± 236 | 15,185 ± 2,205 | 16,676 ± 2,421 | 1,491 ± 217 | 542 ± 79 |

..

Note: ∑Ei: total emissions of six indicators of air pollutants（PM_10_, PM_2.5_, NO_X_, SO_X_, CO, HC）; Mean ± Standard Deviation（M±SD）

Table S6. Mode summary of the multiple linear regression analysis on the relationship between average power consumption by the ship’s generator, sea temperature, wind, and the number of refrigerated containers loaded on the ship.

|  |  | | | | | | | | | |
| --- | --- | --- | --- | --- | --- | --- | --- | --- | --- | --- |
| Mode | | predictor variables | unstandardized coefficient | | R^2^ | R^2^ _adj_ | F | P value ＜0.05  (significant at 5% level) | Collinearity Statistics | |
|  |  |  | Estimated value of ß_0_ | Standard error |  |  |  |  | tolerance | VIF |
| 1 | (Constant) | | 57.831 | 90.455 | 0.768 | 0.766 | 452.396 | 0.524 |  |  |
|  | Number of refrigerated containers loaded | | 4.270 | 0.201 |  |  |  | 0.000 | 1.000 | 1.000 |
| 2 | (Constant) | | -406.732 | 110.882 | 0.815 | 0.815 | 37.090 | 0.000 |  |  |
|  | Number of refrigerated containers loaded | | 4.503 | 0.183 |  |  |  | 0.000 | 0.956 | 1.046 |
|  | sea temperature | | 22.716 | 3.730 |  |  |  | 0.000 | 0.956 | 1.046 |
| 3 | (Constant) | | -389.813 | 107.985 | 0.829 | 0.825 | 8.796 | 0.000 |  |  |
|  | Number of refrigerated containers loaded | | 4.382 | 0.182 |  |  |  | 0.000 | 0.908 | 1.101 |
|  | sea temperature | | 20.837 | 3.682 |  |  |  | 0.000 | 0.928 | 1.078 |
|  | Tailwind or Headwind | | 148.384 | 50.032 |  |  |  | 0.004 | 0.935 | 1.069 |

Table S7. Power consumption and air emissions at each port whether shore power adopted.

| Ports | Generator’s average power consumption  kWh（Mean ± SD） | PM_10_(g h^-1^) | PM_2.5_(g h^-1^) | NO_x_ (g h^-1^) | SO_x_ (g h^-1^) | CO (g h^-1^) | HC (g h^-1^) | ∑Ei (g h^-1^) |
| --- | --- | --- | --- | --- | --- | --- | --- | --- |
| LAX | 0 | 0 | 0 | 0 | 0 | 0 | 0 | 0 |
| OKL | 0 | 0 | 0 | 0 | 0 | 0 | 0 | 0 |
| YTT | 0 | 0 | 0 | 0 | 0 | 0 | 0 | 0 |
| **Average ± SD** | **0** | **0** | **0** | **0** | **0** | **0** | **0** | **0** |
| TCM | 1,651 ± 232 | 71 ± 10 | 79 ±11 | 16,344 ± 2,301 | 27 ± 3 | 2,311 ± 325 | 990 ± 139 | 19,825 ± 2,791 |
| KSG | 1,508 ± 329 | 65 ± 14 | 72 ±15 | 14,928 ± 3,258 | 25 ± 5 | 2,111 ± 460 | 904 ± 197 | 18,108 ± 3,952 |
| HKG | 1,339 ± 13 | 58 ± 0.5 | 64 ± 0.6 | 13,258 ± 132 | 22 ± 0.2 | 1,473 ± 14 | 535 ± 5 | 15,412 ± 154 |
| TPE | 1,442 ± 64 | 65 ± 2 | 69 ± 3 | 14,278 ± 636 | 24 ± 1 | 2,019 ± 90 | 865 ± 38 | 17,319 ± 772 |
| **Average ± SD** | **1,516 ± 249** | **65 ± 10** | **72 ± 11** | **15,007 ± 2,468** | **25 ± 4** | **2,060 ± 421** | **868 ± 204** | **18,100 ± 3,082** |
